# Can Disinfection Robots Reduce the Risk of Transmission of SARS-CoV-2 in Health Care and Educational Settings?

**DOI:** 10.2196/20896

**Published:** 2020-09-15

**Authors:** Kathrin Cresswell, Aziz Sheikh

**Affiliations:** 1 Usher Institute The University of Edinburgh Edinburgh United Kingdom

**Keywords:** robotics, disinfection, SARS-CoV-2, COVID-19, risk, transmission, virus

## Abstract

We explore the opportunities and challenges surrounding the use of disinfection robots to reduce the risk of SARS-CoV-2 transmission in health care and educational settings. Although there is some potential for deploying robots to help with manual cleaning, the evidence base is mixed, and we highlight that there needs to be work to establish and enhance the effectiveness of these robots in inactivating the virus.

SARS-CoV-2 can be transmitted through droplets and contact with contaminated surfaces [1]. To contain the spread, there is a need for more regular and deeper cleaning of indoor surfaces, for example, in schools, care homes, and health care facilities. There is also a need to reduce human exposure to potentially contaminated surfaces. As a result, there is now a greater interest in cleaning and disinfection robots in these settings [2-4]. Such robots are, for example, currently routinely cleaning the Hong Kong metro, and the Smart Field Hospital in Wuhan uses them in an attempt to reduce the spread of SARS-CoV-2 [5,6].

Existing disinfection robots work through a combination of automated or semiautomated processes. They can clean or disinfect floors and surfaces but increasingly focus on disinfecting whole rooms with increasingly complex distribution systems. These most commonly include machines using UV-C light, which works by altering DNA and RNA so that organisms cannot replicate, and vapor and fogging systems that spray chemical disinfectants.

However, despite their increasing use and demand across settings, evidence of their effectiveness is mixed. There is no existing work exploring the effectiveness of disinfection robots in relation to SARS-CoV-2 and other viruses, and the evidence of the impact of UV-C and vapor on health care–associated infections is also limited. In health care settings, both UV-C light and chemical-based disinfection methods (most commonly hydrogen peroxide vapor) do not demonstrate any significant impact on reduced infection rates, although some studies have identified some positive trends and demonstrated a reduction in surface contamination [7-9]. Not surprisingly, UV-C light and chemicals need to touch a surface to be effective, and this may not always be the case—they have issues with shadows, may not reach all areas of concave surfaces, and their effectiveness reduces with distance [10,11]. This work is further complicated by a lack of evidence around how much contamination actually leads to infection and adverse patient outcomes, but there appears to be a general agreement that both techniques are most effective when combined with manual cleaning [12].

Studies investigating cleaning robots using these techniques are limited. The few existing investigations have found that cleaning robots using UV-C light and hydrogen peroxide can deliver some benefits in reducing microbial surface contamination but only when combined with manual cleaning [13,14]. The study quality is relatively low for both applications with possible commercial biases.

Deploying the current generation of cleaning and disinfection robots in health care settings, care homes, and schools is, therefore, unlikely to be of major benefit, and there needs be work to establish and enhance the effectiveness of these robots in inactivating SARS-CoV-2. In addition to concerns around effectiveness, these devices are expensive at between US $30,000 and US $135,000 per unit, and organizations need to train staff to deploy and control them [13,15-17]. Disinfectant chemicals and UV-C light can also be dangerous to human health, so people typically need to leave while the robot cleans the room. This is particularly concerning for communal settings but does not preclude the use of UV-C light in enclosed empty spaces. Other factors to consider include disinfection time (some devices take a few hours per room) and issues with physical spaces and navigation (robots are not good at climbing stairs) [14,18].

Floor cleaning robots are likely to be cheaper units that can relatively easily and quickly be adapted (eg, from other types of service robots) and that can focus on one aspect of the physical environment (ie, the floor) while humans work in parallel with them, eliminating issues around disinfection time. There is, therefore, a need to catalyze the development of floor cleaning robots that can regularly clean communal settings, particularly those with a high risk of transmitting nosocomial infections. These devices can augment manual cleaning, for instance, through supporting an already stretched workforce and through reducing the risk of exposure for cleaning staff and those who work in these settings (eg, doctors, nurses, assistants, teachers), particularly in the context of shortages of personal protective equipment. Some have noted issues with the compliance of cleaning protocols promoting use of these robots, and others have highlighted the importance of effective integration with existing routines and operations [19,20], but this is unlikely to be a significant hurdle in a times of global need. If the current generation of cleaning and disinfection robots are viewed as a panacea to reduce the spread of SARS-CoV-2, the resulting overreliance on their performance may jeopardize lives unnecessarily, but this is an area for urgent development that could help with lockdown exit strategies.
